# Risk of osteoporosis in testicular germ cell tumour survivors: A systematic review of the literature

**DOI:** 10.1002/bco2.183

**Published:** 2022-08-18

**Authors:** Josephina P. M. Vrouwe, Pauline M. L. Hennus, Neveen A. T. Hamdy, Susanne Osanto, Peter‐Paul M. Willemse

**Affiliations:** ^1^ Department of Medical Oncology Leiden University Medical Centre Leiden The Netherlands; ^2^ Centre for Human Drug Research Leiden The Netherlands; ^3^ Department of Urology University Medical Centre Utrecht Utrecht The Netherlands; ^4^ Department of Urology Amphia Hospital Breda The Netherlands; ^5^ Department of Medicine, Division of Endocrinology, and Center for Bone Quality Leiden University Medical Centre Leiden The Netherlands

**Keywords:** bone mineral density, chemotherapy, hypogonadism, osteopenia, osteoporosis, testicular germ cell tumour

## Abstract

**Context:**

Testicular germ cell tumour (TGCT) survivors are potentially at risk of developing osteoporosis, because of increased risk for disturbed bone remodelling associated with hypogonadism and anti‐cancer treatment. A number of studies show bone loss and increased fracture risk in TGCT survivors, but data are scarce. There are no clinical guidelines or recommendations issued to address skeletal health in this group of patients potentially at high risk for osteoporosis.

**Objective:**

To conduct a systematic review of available literature addressing bone health in TGCT patients. Subgroup analysis was performed to identify risk factors for bone loss and increased fracture risk.

**Evidence Acquisition:**

Relevant databases, including MEDLINE, Embase and the Cochrane Library, including all English written comparative studies addressing bone health in TGCT patients, were searched up to December 2021 and a narrative synthesis was undertaken. Risk of bias (RoB) was assessed using Cochrane ROBINS‐I tool.

**Evidence Synthesis:**

Ten studies (eight cross‐sectional and two longitudinal), recruiting a total of 1997 unique TGCT patients, were identified and included in the analysis. Bone health was reported in various ways in different studies, and subgroups were defined heterogeneously, resulting in a widely varying prevalence of osteoporosis of up to 73.2% of patients. Six studies reported low BMD associated with higher luteinizing hormone levels and one study showed a correlation between follow up duration and bone loss.

**Conclusions:**

TGCT survivors are at risk of developing osteoporosis and sustaining fragility fractures. Chemotherapy, pituitary‐gonadal axis dysfunction and ageing are key risk factors, although available data are scarce. With increasing survival of TGCT patients, a clear unmet need has been identified to systematically evaluate and monitor skeletal health in larger numbers of survivors in order to develop best clinical practice guidelines to manage the insidious but potentially preventable and treatable skeletal complications of TGCT.

## 1 INTRODUCTION

Testicular germ cell tumours (TGCTs) are the most common malignancy in men aged 15 to 40 years,^1^, ^2^ representing a global incidence of 552,266 new cases per year in 2012. The introduction of cisplatin‐based chemotherapy in the management of TGCT patients in the seventies that resulted in a significant increase in cure rate to >95%,^1^, ^3^ and thus to a significant increase in survival time allowing the development of late comorbidities of initial disease as well as its treatment such as persistent hypogonadism, cardiovascular disease, metabolic disease and secondary malignancies to be observed after decades of follow up.^4^, ^5^ Depending on disease stage at diagnosis, treatment administered and time elapsed since treatment, between 16% and 27% of TGCT survivors have been reported to be hypogonadal.^6^, ^7^, ^8^ This increased risk for hypogonadism, a recognized significant risk factor for bone loss and increased fracture risk particularly in elderly patients, is possibly exacerbated by the higher prevalence of testicular dysgenesis syndrome observed in TGCT patients.^9^ The cytotoxic chemotherapy and concomitant administration of corticosteroids, which are administered to TGCT patients, have also been associated with Leydig cell insufficiency‐induced hypogonadism,^10^, ^11^, ^12^ and with increased prevalence of low bone mineral density (BMD).^13^ Whether this is a direct effect of chemotherapy on bone remodelling, or an indirect effect on this process due to Leydig cell insufficiency and associated hypogonadism, is as yet to be established.^14^ Whereas a number of studies address bone health in TGCT survivors, outcomes vary widely between different studies.^15^, ^16^ Low BMD is generally expressed as osteopenia, which is a BMD between −1 SD and −2.5 SD below average, and osteoporosis, which represents a BMD −2.5 SD below average healthy young persons. The current EAU germ cell tumour guideline does not address bone health evaluation and monitoring in TGCT survivors.^17^ The reported relatively high prevalence of hypogonadism and potential chemotherapy associated risk for bone loss and increased fracture risk in TGCT survivors has led us to systematically review all available evidence for increased prevalence of osteoporosis and fracture risk in this group of patients.

The main objective of this systematic review was to summarize available literature evidence for bone loss and increased fracture risk and potential risk factors thereof in TGCT survivors, in order to enable the issuing of best clinical recommendations for the evaluation and monitoring of this vulnerable group's bone health.

## 2 METHODS

### 2.1 Search strategy and data sources

The protocol for this review has been published (www.crd.york.ac.uk/PROSPERO; registration number CRD42019119868). Publications from 1990 to December 2021 were searched. The study selection process was done according to the Preferred Reporting items for Systematic reviews and meta‐analyses (PRISMA).^18^

The full search strategy can be found as supporting information.

### 2.2 Inclusion and exclusion criteria

All comparative studies were included. Single‐arm case series, case reports, commentaries, reviews and editorial commentaries were excluded. Relevant systematic reviews were scrutinized for potentially relevant studies for inclusion. Studies had to involve adult men with histologically proven TGCT stages T1–T3 according to the TNM staging system, who were treated with orchidectomy with or without chemotherapy and/or radiotherapy. Comparative arms could consist of healthy adult males, a non‐cancer patient group or different treatment or outcome arms of TGCT patients. Studies that included patients with a metabolic bone disease or congenital hypogonadism were excluded.

Only studies that reported BMD as measured using dual X‐ray absorptiometry (DXA) and/or fracture rates were included.

### 2.3 Data extraction

**FIGURE 1:**
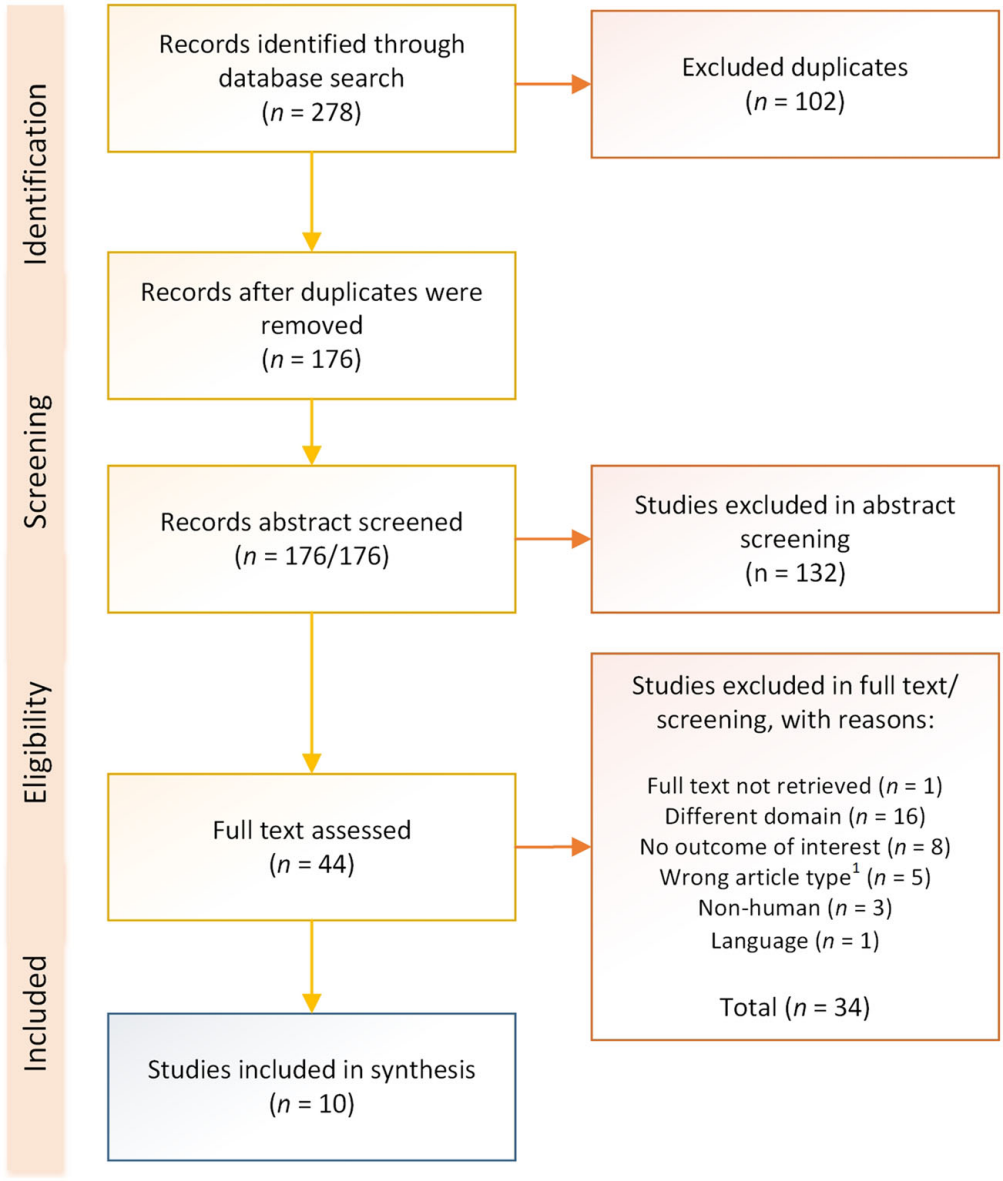
Study selection flow diagram according to the Preferred Reporting items for Systematic reviews and Meta‐Analyses (PRISMA) guidelines. The search was performed in 2019 and updated in December 2021. Legend: ^1^Wrong article types included case reports and reviews.

Two authors (JPMV and PMLH) independently reviewed all titles, article abstracts and full‐text articles for inclusion in the systematic review of the literature. At each step, outcomes were summarized, compared and discussed. Disagreement was resolved by consensus after discussion or consultation with a third reviewer (PMW). The selection process is documented in a Preferred Reporting Items for Systematic Reviews and Meta‐Analyses (PRISMA) flow diagram (Figure 1).^18^ A data extraction form was developed to enable uniform collection of detailed information from the studies that met the inclusion criteria and their outcomes. In case additional data were required to enable comparison with other included papers, authors of the selected articles were approached to request the missing data.

Extracted study characteristics included country of conduct, study objective, study design, outcome measures, sample size (*N*), source of the study population, eligibility criteria, treatment arms and methods, including BMD definition of osteoporosis.

Data extracted also included demographic data (age, follow‐up duration and BMI), details of treatment, BMD measurements (expressed as absolute values in g/cm^2^, T‐scores and Z‐scores), plasma measurements of gonadal hormones and bone status indicators and any fracture data if available. In case of longitudinal studies, both baseline and follow‐up data were extracted if available.

### 2.4 Assessment of risk of bias

The risk of bias of each included study was independently assessed by two authors (JPMV, PMLH) using the Cochrane ROBINS‐I tool.^19^ Any disagreement was resolved by consensus after discussion or consultation with a senior reviewer (PMW). A list of outcome‐specific prognostic confounders was a priori defined by the authors for each domain. These confounders included age, tumour type, follow‐up duration, definition of the intervention, missing data across groups and incomplete reporting of results.

### 2.5 Data analysis and statistics

A narrative synthesis of the included studies was performed using descriptive statistics to summarize study and patient characteristics. Subgroups were defined on the basis of treatment administered, gonadal status, prevalence of fractures and follow‐up duration. In case of longitudinal studies, baseline and follow‐up data were included in the evaluation.

Outcome of laboratory investigations of gonadal hormones and/or bone status indicators, fracture rates and fracture risk scores (e.g., FRAX‐score) were analysed and reported in a descriptive manner.

## 3 RESULTS

### 3.1 Study selection

The PRISMA flow chart depicting the process of the systematic literature search and selection of the included studies is shown in Figure 1.^18^

After exclusion of duplicate studies, two authors (JPMV and PMH) selected 44 articles for full‐text evaluation after independently completing a review of 176 titles and abstracts. A final cross‐checked selection was made in keeping with the outlined inclusion criteria for the review. This selection resulted in the inclusion of 10 full‐text publications, providing data on a total of 2921 TGCT patients, 1997 TGCT patients after confirmation of uniqueness. A combined total of 180 non‐TGCT subjects were included as controls across the 10 studies.

### 3.2 Characteristics of the studies included in the systematic review

Of the 10 studies fulfilling the inclusion criteria for the systematic review, two were prospective non‐randomized controlled studies (Willemse 2014, IJpma).^20^, ^21^ The others were cross‐sectional, non‐randomized controlled studies.^15^, ^16^, ^21^, ^22^, ^23^, ^24^, ^25^, ^26^, ^27^ Population sizes ranged from 30 to 1249 patients. Study characteristics of the included studies are shown in Table 1.

**TABLE 1:** Summary of study and patient characteristics

Study ID	Country, design, recruitment period	Study arms	Treatment arms	*N*	Age, mean, (SD), [range]	Follow‐up in years, mean, (SD), [range]	BMI, mean, (SD), [range]	Primary objective of the study
**Ondrusova (2018)** ^26^	Slovakia, Cross‐sectional, 2005–2015	Long‐term TGCT survivors	Full group	1249	39	7 (7.2)		Evaluate effects of different therapeutic approaches for TGCT and changes in sex hormone levels and their impact on BMD.
			
			OE	313	38	6 (7.5)		
			OE + CT	665	41	5 (6.4)		
			OE + RT	271	38	7 (7.4)		
**IJpma (2017)** ^21^	The Netherlands, Cross sectional, 2012–2015	TGCT	baseline CT ([B]EP)	21	32 [27–36]	1	24.3 (22.2–26.4)	Investigate systematic pattern of changes in taste and smell function, food preference, dietary intake and body composition can be found and persists over time in TGCT patients treated with cisplatin‐based CT.
					
					
		TGCT	1 month after CT ([B]EP)	11				
		TGCT	1 y after CT ([B]EP)	7				
		Healthy controls	N/A	48	32 [29–36]	N/A	23.5 (21.7–25.8)	
**Isaksson (2017)** ^24^	Sweden, Cross‐sectional, 2001–2006	TGCT	Full group	89	40.3 (7.4)	9.3 (2.69)	26.7 (3.84)	To assess low BMD, the risk of low BMD and the possible associations with biochemical signs of hypogonadism and cancer treatment given.
		TGCT	OE	11	37.0 (7.4)	6.76 (2.47)	26.6 (4.2)	
		TGCT	OE + 1–2 cycles CT	28	28.9 (7.6)	8.60 (2.83)	26.7 (3.4)	
		TGCT	OE + 3–4 cycles CT	23	38.8 (7.1)	10.1 (2.21)	24.9 (2.8)	
		TGCT	OE + > 4 cycles CT	5	40.9 (8.9)	9.68 (2.23)	27.5 (1.3)	
		TGCT	OE + RT	22	45.1 (5.7)	10.3 (2.43)	28.6 (4.8)	
		Healthy controls	N/A	91	41.2 (7.3)	N/A	25.6 (3.3)	
**Willemse (2014)** ^20^	The Netherlands, Prospective follow‐up, 2007–2009	TGCT patients (seminoma and non‐seminoma) treated and disease‐free >3 years after the end of treatment.	Full group	63	33 [16–70]	‐		To evaluate longitudinal changes in BMD in newly diagnosed and recently orchiectomized TGCT patients up to 5 years after anticancer treatment.
			Stage I	27	35 [22–70]	0		
			Stage I 5y F‐U	27		5		
			Disseminated (CT)	36	34 [16–59]	0		
			Disseminated (CT) 5y F‐U	36		5		
**Foresta (2013)** ^23^	Italy, Cross‐sectional, 2010–2011	Testicular germ cell tumours	OE, RT and/or CT	125	34.0 (6.1)	4.6 (2.0)	23.6	To determine bone metabolism markers and BMD in a cohort of normo‐testosteroniemic patients who underwent unilateral OE for TGCT.
		Sexual dysfunction controls	N/A	41	35.8 (6.2)	N/A	22.9	
**Willemse (2010)** ^15^	The Netherlands, Cross‐sectional	Orchiectomized patients with/without CT.	Full group	244	39.4 [18.2–66.9]			To assess skeletal fragility in a cohort of TGCT patients who have been followed‐up for up to 28 years after initial diagnosis and treatment.
		1–28 y after cure (OE and when required CT)	Long term follow‐up group	199	40.0 [18.2–66.9]	[1–28]		
			Long term OE + CT	152				
			Long term OE	47				
		After unilateral orchidectomy, before commencing CT	Newly diagnosed	45^a^	32.0 [18.3–54.3]	0–3 months after OE		
**Murugaesu (2009)** ^16^	United Kingdom, Cross‐sectional, NR	TGCT	Full group	39	48.0 [30–74]	15.7 [5.3–28.3]	24.8 (15.7–35.1)	To establish the long‐term incidence of osteoporosis following OE with or without CT.
		TGCT	OE	14	50.4 [30–74]	13.1 [5.7–23.0]	24.6 (15.7–35.1)	
			OE + CT	25	43.6 [34–64]	17.1 [5.3–28.3]	26.1 (20.6–31.1)	
**Ondrusova (2009)** ^25^	Slovakia, Cross‐sectional, 2005–2009	TGCT	Full group	879	32.6	8 [0.25–38.5]		To investigate hormonal profile and osteological examination in patients with unilateral and bilateral TGCT and come to an algorithm of follow‐up for these patients.
		Unilateral TGCT		823	32 (9.0) [14–68]	7.4 [0.25–29.41]		
			OE + CT					
			OE + RT					
			OE + CT + RT					
			RT in total					
			CT in total					
		Bilateral TGCT		56	41.3	14.6 [1.1–38.5]		
			OE + CT					
			OE + RT					
			OE + CT + RT					
**Brown (2006)** ^22^	United Kingdom, Cross‐sectional, 2001–2003	TGCT	OE	101	42.3 [23.6–69.6]	N/A	NR	To assess the extent of bone loss due to previous CT in men, and to determine if the rate of bone turnover in such patients is abnormal by measurement of bone metabolism markers.
		TGCT	OE + CT	64	40.4 [19.4–67.8]	4.1 [1.0–29.2]	NR	
**Stutz (1998)** ^27^	United Kingdom, Cross‐sectional, 1994–1995	Intra‐patient comparison of TGCT patients	Full group	30	42.93, (9.82), [25–63]	2.3 [0.17–10.5]		To determine whether treatment of TGCT with RT results in significant long‐term effects on BMD.
			irradiated side	30	42.93 (9.82) [25–63]	2.3 [0.17–10.5]		
			non‐irradiated side	30	42.93 (9.82) [25–63]	2.3 [0.17–10.5]		

Abbreviations: AF, alkaline phosphatase; BMD, bone mass density; BMI, body mass index; Ca, calcium; CT, chemotherapy; CTx, C‐telopeptide; DXA, dual energy Xray absorptiometry; FSH, follicle stimulating hormone; LH, luteinizing hormone; LS, lumbar spine; NR, not reported; OE, orchiectomy; Oes, estradiol; prox., proximal; PTH, parathyroid hormone; RT, radiotherapy; SD, standard deviation; SHBG, sex hormone binding globuline; T, testosterone; TGCT, testicular germ cell tumour; Vit. D, vitamin D; WHO, World Health Organization.

^a^ Short‐term follow‐up group excluded from BMD analysis, as these were the same patients as those analysed in the Willemse (2014) study.

^b^ Data from Ondrusova (2009) is not interpreted separately, as it appears that there is a large overlap with the population of Ondrusova (2018).

Within studies, patients were grouped based on treatment received,^15^, ^22^, ^24^, ^26^, ^27^ tumour stage (Murugaesu, Willemse 2014, Ondrusova, 2009),^16^, ^20^, ^25^ or presence of vertebral fractures on routine spine X‐rays (Willemse, 2010).^15^ Three studies compared TGCT patients with a control group of men without a diagnosis of cancer. Two of these three studies included healthy controls (IJpma, Isaksson), and the third included patients with sexual dysfunction as control group (Foresta).^10^, ^21^, ^23^ Nine studies additionally reported plasma gonadal hormone levels of LH, FSH, testosterone, SHBG and estradiol levels.^15^, ^16^, ^20^, ^21^, ^22^, ^23^, ^24^, ^25^, ^26^ Bone status indicators were reported in four studies, of which vitamin D, calcium and parathyroid hormone were reported in two or more studies.^15^, ^16^, ^22^

### 3.3 Risk of bias assessment

**FIGURE 2:**
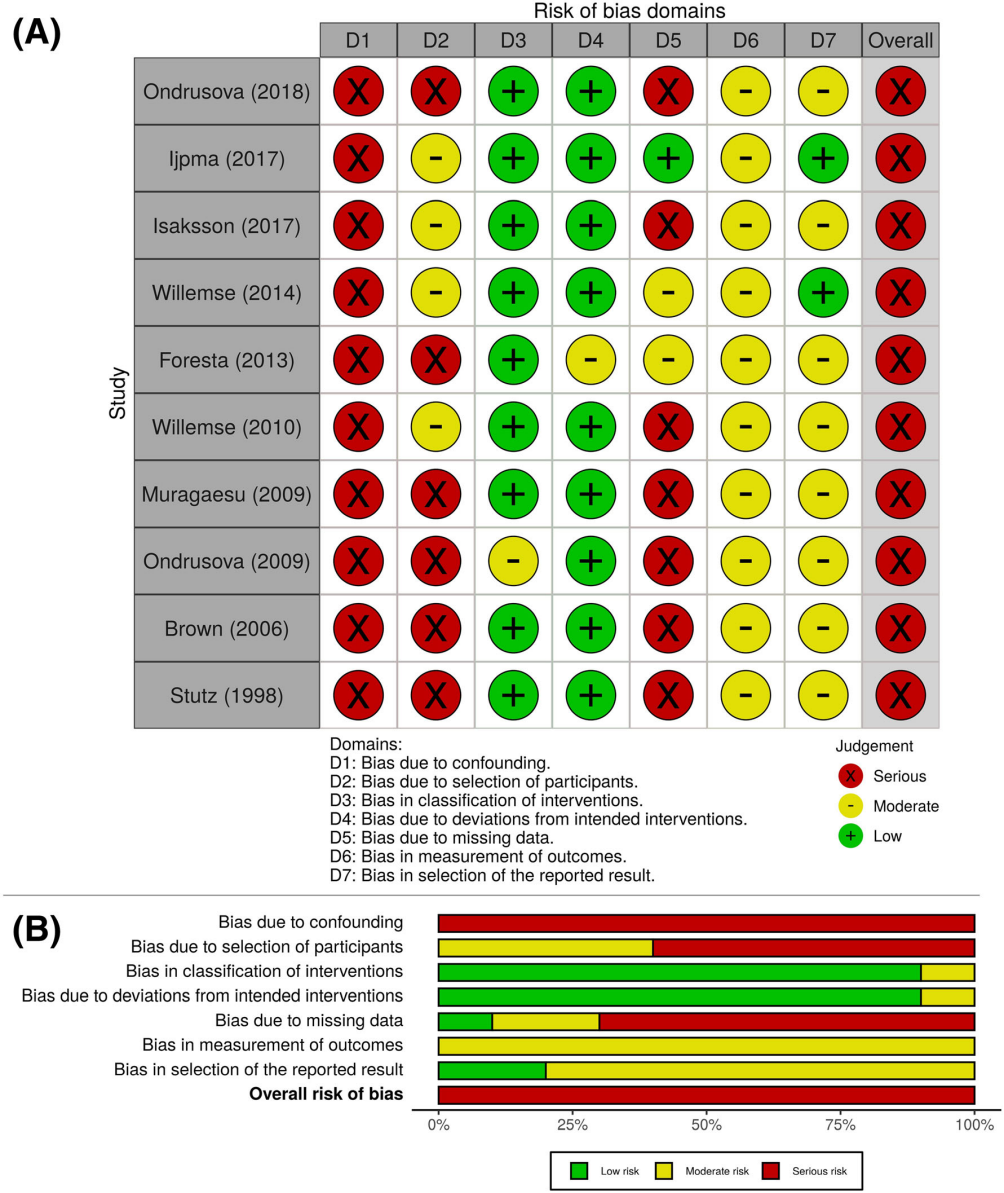
Risk of bias assessment for (A) individual studies and (B) across studies. Legend: Based on the assessment of each domain, domain‐level risk‐of‐bias judgement are ‘low’: comparable to a RCT with regard to this domain (green); ‘moderate’ sound for a non‐randomized study with regard to this domain but cannot be considered comparable to a well‐performed randomized trial (yellow); ‘serious’: the study has some important problems in this domain (red); ‘critical’ the study is too problematic to provide any useful evidence and should not be included in any synthesis. The overall risk of bias is determined based on the assessment of all domains; as all studies had at least one domain with serious risk of bias (and none with a critical risk of bias), all studies must be assessed as having serious risk of bias.^19^

The RoB assessment for all included studies is shown in Figure 2. This risk was ‘serious’ in all studies, although its potential cause remained confounding as treatments were used to define groups. There was also a potential bias in the selection of participants due to missing inclusion or exclusion criteria.

### 3.4 BMD measurements

The DXA systems used, the sites measured and the definitions used to interpret measurement outcomes are shown in Tables 1 and 2. The systems applied were the Horizon Hologic (six studies), Lunar Progidy (three studies) or not reported (one). All studies reported at least lumbar spine BMD outcomes. The expression of outcome measures for BMD varied between studies between T‐ and/or Z‐scores, absolute BMD in g/cm^2^, or odds ratios (OR) for osteopenia and osteoporosis compared to a reference group.

Nine studies referred to the world health organization (WHO) definitions for osteopenia (T‐score > −1 to ≤ −2.5) and osteoporosis (T‐score ≤ −2.5).^15^, ^16^, ^20^, ^21^, ^22^, ^24^, ^25^, ^26^, ^27^ Foresta et al. did not provide the criteria used to define osteoporosis or osteopenia.^23^ The prevalence of osteoporosis and/or osteopenia was reported in eight papers.^15^, ^20^, ^22^, ^23^, ^24^, ^25^, ^26^, ^27^

### 3.5 Treatment groups

Seven studies compared orchiectomy‐only treated patients with patients who were treated with orchiectomy and with chemotherapy and/or radiotherapy.^15^, ^16^, ^20^, ^22^, ^24^, ^25^, ^26^ Isaksson also compared the outcomes in different TGCT treatment groups with those of healthy men.^24^ Foresta bundled all treatment groups and compared those with the results of a non‐TGCT group.^23^ Two studies only included patients who had a specific treatment combination: IJpma et al. compared patients who had orchiectomy and chemotherapy with healthy subjects, and Stutz et al. performed a within‐patient comparison of patients' irradiated and non‐irradiated sides.^21^, ^27^

### 3.6 BMD results

Table 2 details BMD results for all 10 studies included in the systematic review.

**TABLE 2:** Summary of bone mineral density outcomes

Study characteristics			Lumbar spine BMD outcomes^**a**^			Proximal femur/Total hip BMD outcomes^**a**^			Other BMD outcomes
Study ID	Treatment arms	DXA Imaging device	BMD g/cm^2^ mean (SD)	T‐Score (SD) <IQR> [range]	Z‐score (SD) <IQR> [range]	BMD g/cm^2^ (SD) <IQR> [range]	T‐Score (SD) <IQR> [range]	Z‐score (SD) <IQR> [range]	
**Ondrusova (2018)** ^26^	TGCT full group	Hologic							Osteopenia/osteoporosis in 136 (43.45%) patients. Osteopenia/osteoporosis in 298 (44.81%) patients, OR 1.233, 95%CI 0.885–1.717. Osteopenia/osteoporosis in 139 (51.29%) patients, OR 1.018, 95%CI 0.775–1.338. *NS difference between patient groups, p values not reported*.
	TGCT OE								
	TGCT OE + CT								
	TGCT OE + RT								
	
**IJpma (2017)** ^21^	TGCT baseline CT	Hologic			−0.2 < −0.8–1.6>				
	TGCT 1 month after CT			−0.5 < −1.3–0.4>					
	TGCT 1 y after CT			−0.5 < −0.9−−0.4>					
	Healthy controls			‐0.4 < −1.2–0.6>					
			*Lower BMD in patients at follow‐up compared to baseline (1 m p = 0.010 and 1 y p = 0.034)*						
**Isaksson (2017)** ^24^	TGCT Full group	Lunar	1.248 (0.162)		1.248 (0.162)	1.073 (0.129)		−0.119 (0.934)	Low BMD (Z‐score < −1) in 19% (hip) and 21% (LS) of the patients and in 12% (hip) and 26% (LS) of the control group (NS difference).
	TGCT OE		1.275 (0.137)		0.242 (0.913)	1.127 (0.119)		0.294 (0.768)	
	TGCT OE + 1–2 cycles CT		1.241 (0.173)		−0.141 (1.40)	1.084 (0.145)		−0.104 (1.039)	Subanalyses of hypogonadal versus eugonadal TGCT patients: Patients with treated or untreated hypogonadism had lower hip BMD. Eugonadal patients: mean 1.081 g/cm^2^, (SD 0.121), untreated hypogonadal patients: mean: 1.066 g/cm^2^ (SD 0.167), *p =* 0.037, treated hypogonadal patients: mean 1.044 g/cm^2^ (SD 0.084), *p =* 0.043. Patients with untreated hypogonadism had lower LS BMD compared to eugonadal patients. Eugonadal patients: 1.268 g/cm^2^ (SD 0.154); Untreated hypogonadal patients: mean:1.207 g/cm^2^ (SD 0.198), *p* = 0.022, Treated hypogonadal patients: mean 1.206 g/cm^2^ (SD 0.125), *p* = 0.012. Absolute BMD and Z‐scores of the hip did not differ between irradiated and the non‐irradiated sides (both *p* = 0.37).
	TGCT OE + 3–4 cycles CT		1.233 (0.121)		0.004 (0.930)	1.022 (0.079)		−0.416 (0.618)	
	TGCT OE + > 4 cycles CT		1.139 (0.060)		−1.226 (0.442)	1.012 (0.071)		−0.783 (0.609)	
	TGCT OE + Irradiation		1.276 (0.208)		0.141 (1.64)	1.092 (0.155)		0.058 (1.110)	
	Healthy controls		1.206 (0.159)		−0.230 (1.23)	1.082 (0.125)		0.038 (0.867)	
			*NS difference between treatment groups (p value range: 0.23–0.67). NS difference between full group of TGCT patients and Healthy controls (p = 0.27)*			*NS difference between treatment groups (p value range: 0.07–0.51), lowest p values in CT groups. NS difference between full group of TC patients and Healthy controls (p = 0.14)*			
**Willemse (2014)** ^20^	TGCT full group	Hologic							Prevalences at baseline Osteoporosis: 3.2% at LS, 0% at the hip Osteopenia: 9.5% at LS and hip, 14.3% at LS and 1.6% at the hip. NS difference between metastatic or Stage 1 TGCT patients. Prevalences at 1 y after anticancer treatment: Osteoporosis: 1.6% at LS, 0% at the hip. Osteopenia: 12.7% at LS and hip, 20.6% at LS, 1.6% at the hip. NS difference between metastatic‐ or Stage 1 TGCT patients. BMD changes were independent of gonadal state, vit. D and β‐CTX
	TGCT Stage I (OE)			−0.21, 95%CI—2.42–2.97	−0.14, 95%CI—2.42–2.97		0.02, 95% CI—1.42–1.53	0.23, 95%CI—1.42–1.57	
	TGCT Stage I (OE) 5y F‐U				−0.74, 95% CI—2.57–3.55			−0.35, 95%CI—1.60–1.09	
	TGCT Disseminated (OE + CT)			−0.43, 95%CI—2.87–1.78	−0.37, 95% CI—2.54–1.78		0.02, 95%CI—1.49–1.77	0.22, 95% CI—1.12–1.90	
	TGCT Disseminated (OE + CT) 5y F‐U				−0.61, 95%CI—2.38–1.64			−0.22, 95%CI—1.23–1.09	
			*NS difference between groups at baseline. Decreased BMD at 5 years in patients with metastatic disease and CT (p < 0.004)*			*NS difference between groups at baseline. Decreased BMD at 5 years in patients with metastatic disease and CT (p < 0.0001)*			
**Foresta (2013)** ^23^	TGCT OE, RT and/or CT	NR	1.003 (0.146)			0.981 (0.115)			Low BMD (Z‐score <‐2SD) in 23.8% of patients with TGCT, compared to 0% in the sexual dysfunction group (*p* < 0.0005) Higher prevalence of low BMD was found in patients with longer F‐U. The patient groups were divided in subgroups with a F‐U duration of 2–3 y (36 subjects), 4–5 y (42 subjects), 6–7 y (27 subjects) from OE and low BMD was found in, respectively, 16.6% (6/36), 16.7% (7/42) and 40.7% (11/27) of patients; 6–7 y: *p* < 0.05 versus 2–3 and 4–5 y groups.
	Sexual dysfunction		1.179 (0.119)			1.151 (0.128)			
			*Lower BMD in TGCT patients (p < 0.00001)*			*Lower BMD in TGCT patients (p < 0.00001)*			
**Willemse (2010)** ^15^	TGCT full group	Hologic							Osteoporosis in 5.5%, Osteopenia in 41.7%
	TGCT 1–28 y follow‐up			−0.33 (1.19)	−0.14 (1.16)		−0.53 (0.93)	−0.05 (0.89)	Z‐scores between −1.0 and −2.0 in 26.1% and Z‐scores <−2 in 7.0 (femoral neck, LS or both sites).
	TGCT, VF								
	TGCT, no VF			−0.33 (1.32)	−0.17 (1.35)		−0.32 (SD 0.96)	0.13 (0.95)	NS difference in the prevalence of osteoporosis between treatment groups. Severity or number of VF was independent of age, tumour type, staging, previous CT, gonadal status, vitamin D levels or BMD values.
*Additional data long term F‐U*	TGCT OE		1.05 (0.145)			0.888 (0.13)			
	TGCT OE + CT		1.04 (0.15)			0.858 (0.13)			
			*NS difference between groups with or without VF, and treatment groups*.			*NS difference between groups with or without VF, and treatment groups*.			
**Murugaesu (2009)** ^16^	TGCT OE with or without CT	Hologic		0.1, 95%CI—0.3–0.5	0.4, 95%CI—0.1–0.8		−0.1, 95% CI—0.4–0.2	0.3, 95%CI—0.001–0.6	Neither OE nor OE + CT predisposed to osteoporosis.
	Local disease, OE			0.2, 95%CI—0.3–0.7	0.5, 95%CI—0.1–1.1		−0.1, 95% CI—0.6–0.3	0.2, 95%CI—0.2–0.7	There was no evidence of an association between low BMD and length of F‐U, as assessed by logistic regression (*p* value not reported*)*
	N+/M + disease, OE + CT			−0.1, 95%CI—0.8–0.6	0.1, 95%CI—0.7–0.8		−0.1, 95% CI—0.03–0.5	0.4, 95%CI—0.1–0.8	
			*NS difference between patient groups, T‐score: p = 0.48, Z‐score: p = 0.37*.			*NS difference between patient groups, T‐score: p = 0.50, Z‐score: p = 0.54*.			
**Ondrusova (2009)** ^25^	TGCT full group	Hologic							‐
	Unilateral TGCT								Osteoporosis and/or osteopenia in 404 patients (49.1%) OR compared to OE alone (95% CI):
	OE + CT								OR osteopenia: 1.19 (0.85–1.66) OR osteoporosis: 1.12 (0.66–1.91)
	OE + RT								OR osteopenia: 1.16 (1.01–1.80) OR osteoporosis: 1.27 (0.67–2.43)
	OE + CT + RT								OR osteopenia: 2.38(0.69–8.17) OR osteoporosis: 1.52 (0.30–7.69)
	RT in total								OR osteopenia: 1.23 (1.02–1.89 OR osteoporosis: 1.30 (0.69–2.44)
	CT in total								OR osteopenia: 1.21 (0.87–1.69) OR osteoporosis: 1.13 (0.67–1.92)
	Bilateral TGCT								Osteoporosis/osteopenia in 41 patients (73.2%) odds ratio for Osteoporosis + osteopenia: 2.57 (95% CI: 1.42–5.02, *p* < 0.001) OR for Osteoporosis compared to unilateral disease: 3.34 (95% CI: 1.44–7.31, *p* < 0.001)
	OE + CT								OR osteopenia: 1.81 (0.39–8.48) OR osteoporosis: 1.23 (0.27–5.65)
	OE + RT								OR osteopenia: 0.76 (0.14–4.16) OR osteoporosis: 0.86 (0.13–5.63)
	OE + CT + RT								not evaluated due to sample size
									*Higher OR for osteoporosis and osteopenia in the bilateral group than the unilateral group (p < 0.001). Higher prevalence of osteopenia/osteoporosis in the unilateral RT treated group (p < 0.05), Otherwise no statistically significant differences between treatment groups*.
**Brown (2006)** ^22^	TGCT OE	Lunar	1.336 (0.185)			1.142 (0.158)			Prevalence of low BMD in OE group: osteopenia: 16.7%, osteoporosis: 0%
	TGCT OE + CT		1.335 (0.153)			1.152 (0.146)			Prevalence of low BMD in OE + CT group: osteopenia: 20.0%, osteoporosis: 1.7%
			*NS difference, (p = 0.680)*			*NS difference, (p = 0.662)*			*p* value not reported BMD was not lower than that of the Lunar reference population.
**Stutz (1998)** ^27^	TGCT survivors, intra‐patient comparison	Lunar	1.290 (0.207)	0.412 (1.725)	0.761 (1.659)	1.09 (0.19)	0.16 (1.2)	0.43 (1.23)	Low BMD: osteoporosis of the LS in 13.3% of patients none had osteopenia of the LS. However, mean Z‐scores of the whole body resulted in a Z‐score significantly greater than 0 (*p* = 0.004). No fractures occurred in the osteopenic patients (*n* = 4)
	irradiated side					1.458 (0.21) [1.099–1.867]			
	non‐irradiated side					1.454 (0.21) [1.025–1.941]			No association of LS T‐score with age was found.
			*t test against mean of 0, BMD Z‐score significantly higher than reference population (p = 0.018)*.			*NS difference between the irradiated and non‐irradiated side (p = 0.855)*			

Abbreviations: 95%CI, 95%‐confidence interval; BMD, bone mineral density; CT, chemotherapy; F‐U, follow‐up; IQR, interquartile range; LS, lumbar spine; NS, non‐significant; N+ M+, disease patients with tumour‐positive lymph nodes or metastatic diseaseOE, orchiectomy; OR, odds ratio; RT, radiotherapy; SD, standard deviation; TGCT, testicular germ cell tumour; VF, vertebral fractures; y, years.

^a^ Different DXA systems use different ethnicity reference populations to calculate T‐ and Z‐scores. For this and various other reasons, outcomes are not directly comparable.

Three studies compared BMD results of TGCT patients who had undergone various treatments with those of non‐TGCT patients. IJpma and Isaksson had healthy controls as control group and Foresta had sexual dysfunction patients as a control group. IJpma and Foresta found a significantly lower BMD at the lumbar spine in TGCT patients compared to controls, with *p* values of *p* < 0.0001, and *p* = 0.010. Foresta also reported a significantly higher prevalence of Z‐scores of ≤−2 in 23.8% in its TGCT group compared to 0% in the control group (*p* < 0.0005).^23^

The third study, by Isaksson et al., had a healthy control group and expressed BMD results as Z‐scores. Although patients treated with chemotherapy had a trend for lower BMD, this was not statistically significant compared to any other TGCT treatment group or healthy controls.^24^

Seven studies evaluated BMD outcomes in TGCT patients treated with orchiectomy alone compared to TGCT patients who had chemotherapy and/or radiotherapy in addition to orchiectomy. IJpma and Willemse (2014) were longitudinal studies and reported a lower BMD in their chemotherapy‐treated group at follow‐up.^20^, ^21^ Ondrusova (2009) reported a higher prevalence of osteoporosis or osteopenia (73.2%) in the patients who had underwent bilateral orchidectomy compared to the unilateral group (49.1%, *p* < 0.001).^25^ Other studies did not report statistically significant differences in BMD at the lumbar spine or hip/proximal femur regions between treatment groups.^15^, ^16^, ^22^, ^24^, ^26^

A within‐patient comparison of BMD at irradiated compared to non‐irradiated hip sites was conducted by Isaksson and Stutz.^24^, ^27^ Both found that the proximal femur BMD was not affected by radiotherapy (*p =* 0.855, *p* = 0.37). Stutz et al. assessed BMD at the lumbar spine in irradiated patients and found that 13.3% had osteoporosis at lumbar vertebrae within the irradiated area, although on average lumbar spine BMD was higher than that of the device's reference population (*p* = 0.018).^27^

### 3.7 Fractures

Fracture related outcomes (vertebral, hip or non‐vertebral) were reported only by Willemse (2010) and Stutz. Stutz reported ‘no fractures’ in the four patients diagnosed with osteoporosis.^27^ In contrast, the study by Willemse (2010) reported a high prevalence of radiological vertebral fractures in 14% of patients based on evaluation of systematically performed lateral X‐rays of the thoracic and lumbar spine in all patients included in their study (*n* = 244), although they found no association between number or grade of severity of vertebral fractures and BMD, age, tumour stage, treatment with chemotherapy, gonadal status or vitamin D levels.^27^

### 3.8 Follow‐up data

In the eight studies with a cross‐sectional design, interval time between treatment administration and analysis of follow‐up data varied from 5 to 28 years after treatment.^15^, ^16^, ^22^, ^23^, ^24^, ^25^, ^26^, ^27^ The longitudinal studies reported follow‐up data for 1 year (IJpma) and 5 years (Willemse, 2014) after start of treatment.

The effects of follow‐up duration on changes in BMD were reported in five studies, with low BMD more frequently found in patients with a longer follow‐up.^16^, ^20^, ^21^, ^23^, ^25^ Foresta reported a Z‐score of ≤−2 in 16.6% of patients after 2–3 years, and in 40.7% at 6–7 years, *p* < 0.05.^23^ Ondrusova found a significant risk of developing osteopenia and/or osteoporosis 8 to 10 years after orchiectomy.^25^ The studies with a longitudinal design by Willemse (2014) and IJpma, found a significantly lower BMD (*p* ≤ 0.004, *p* = 0.034, respectively) at follow‐up than at baseline in patients who had undergone chemotherapy.^20^, ^21^ Murugaesu did not find significant differences in BMD based on follow‐up duration.^16^

### 3.9 Laboratory markers of gonadal status and bone status

Details of plasma levels of gonadal hormones and bone status indicators are shown in Table 3. Plasma levels of luteinizing hormone (LH) and free testosterone (FT) were reported in nine studies, of which Foresta et al. excluded hypogonadal patients.^15^, ^16^, ^20^, ^21^, ^22^, ^23^, ^24^, ^25^, ^26^ None of the studies reported testosterone/LH ratios and six of the nine studies did mention the use of testosterone replacement therapy.^16^, ^21^, ^22^, ^23^, ^25^, ^26^, ^27^ Of the three studies that did, Isaksson did take into account testosterone and LH levels and use of hormone replacement therapy to define hypogonadism and found that hypogonadal patients with and without androgen replacement therapy had 6%–9% lower hip BMD (*p =* 0.043 and *p* = 0.037, respectively).^24^ In the other two studies, by Willemse (2010, 2014), LH levels were not taken into account to define hypogonadism and there was no relationship identified between hypogonadism and BMD.^15^, ^20^

**TABLE 3:** Summary of serum blood measurements

Study characteristics				Sex hormones					Bone markers		
Study ID	Study arms	Treatment arms	*N*	LH (IU/L)^b^mean/median, (SD), <IQR>, [RANGE]	Testosterone^c^ (nmol/L) mean/median, (SD), <IQR>, [RANGE]	FSH (IU/IL)^d^ mean/median, (SD), <IQR>, [RANGE]	Estradiol^e^ (pmol/L) mean/median, (SD), <IQR>, [RANGE]	SHBG^f^ mean/median, (SD), <IQR>, [RANGE]	Vit. D (nmol/L) mean/median, (SD), <IQR>, [RANGE]	Calcium mean/median, (SD), <IQR>, [RANGE]	PTH mean/median, (SD), <IQR>, [RANGE]
**Ondrusova (2018)** ^26^	TGCT	Full group	1249								
		OE	313	Elevated in 23 patients	decreased in 46 patients						
		OE + CT	665	Elevated in 154 patients, OR 2.257 (1.32–3.86)	decreased in 103 patients, OR 1.646 (1.073–2.523)						
		OE + RT	271	Elevated in 43 patients OR 3.79 (2.39–6.02)	decreased in 66 patients, OR 1.050 (0.716–1.539)						
				*Elevated in OE + CT and OE + RT groups*	*NS difference*						
**IJpma (2017)** ^21^	TGCT	baseline CT ([B]EP)	14/15	3.9 <0.3–5.7>	19.0 <4.2–21.0>						
		1 m after CT ([B]EP)	12/17	8.8 <7.1–11.1>	21.5 <15.9–26.56>						
		1 year after CT ([B]EP)	6/7	5.5 <4.5–9.7>	16.3 <12.0–22.4>						
	Healthy controls		46	3.5 <2.8–4.9>	24 <19–28>						
				*p values NR*	*Lower in patients at baseline (p = 0.007)*						
**Isaksson (2017)** ^24^	TGCT	Full group	91	5.1 <3.0–7.0>	12.8 (3.5)			31.3 (11.7)			
		OE	11	3.8 <3.2–4.6>	13.0 (3.9)			28.9 (13.2)			
		OE *+* 1–2 cycles CT	28	5.1 <4.1–6.4>	13.1 (3.7)			28.3 (11.0)			
		OE *+* 3–4 cycles CT	23	6.2 <4.6–8.0>	12.9 (3.6)			31.8 (8.5)			
		OE *+* >4 cycles CT	5	n.d. due to group size	n.d. due to group size			n.d. due to group size			
		OE + RT	22	4.6 <3.0–7.0>	12.4 (3.1)			35.3 (14.1)			
	Healthy controls	N/A	91	3.3 <2.1–4.2>	13.9 (4.0)			31.5 (13.0)			
				*Significantly Elevated in all but the OE only group*.	*NS difference*						
**Willemse (2014)** ^20^	TGCT	full group	63								
		Stage I	27	7.5 (4.9) [2.9–25.6]	17.4 (7.5) [0.2–33.9]	12.4 (7.7) [4.6–32.1]	69 (20) [28–98]	30.5 (18.3) [15.7–48.4]			
		Stage I 5y FU	27	6.6 (4.6) [3.1–20.1]	16.1 (7.2) [0.7–29.2]	11.7 (11.1) [4.5–48.5]	66 (20) [38–99]	33.1 (10.1) [12.5–49.9]			
		Disseminated	36	5.9 (5.8) [0.1–24.8]	17.4 (5.6) [4.7–30.2]	10.4 (11.1) [0.1–44.5]	104 (56) [31–2400]	31.2 (12.0) [6.8–64.0]			
		Disseminated 5y FU	36	6.7 (3.2) [2.1–13.5]	16.2 (5.8) [7.0–29.5]	12.8 (8.0) [2.9–29.1]	82 (21) [35–118]	32.8 (13.9) [11.7–59.3]			
				*Higher in patients with disseminated than stage 1 disease (p < 0.001)*	*NS*	*Higher in patients with disseminated than stage 1 disease (p = 0.008)*	*Higher in patients with disseminated than with stage 1 disease (p = 0.007)*	*NS*			
**Foresta (2013)** ^23^ ^a^	TGCT	OE, RT and/or CT	105	6.9 (3.6)	17.6 (4.9)	12.5 (9.9)	95.4 (33.9)		41.6 (20.6)	2.41 (0.11)	72.8 (28.6)
	Sexual dysfunction	N/A	41	3.9 (2)	16.6 (5.7)	3.6 (1.6)	89 (32)		74.9 (3.9)	2.38 (0.1)	49.5 (14.2)
				*(p < 0.00001)*	*NS*	*p < 0.00001*	*NS*		*(p < 0.00001)*	*NS*	*(p < 0.00001)*
**Willemse (2010)** ^15^	TGCT	full group	279	6.0 [0.1–43.5]	15 [7–34]	12 [0.1–100.1]	76 [4–373]		59 [18–149]	2.45 [2.00–2.83]	5.1 [0.6–19.0]
		Long term follow‐up group	254	high LH: 36 (18.1%)	15 [7–34]		decreased in 91 (45.7%)				
		Long term follow‐up with VF	27	7.4 [2.8–19.7]	15 [7–26]	11 [5.3–39.0]	71 [48–134]		60 [27–116]	2.44 [2.00–2.66]	5.9 [2.1–10.8]
		Long term follow‐up without VF	172	6.1 [1.9–37.5]	14 [7–32]	13.5 [2.4–80.0]	74 [10–187]		60 [20–149]	2.45 [2.24–2.83]	4.9 [0.6–19.0]
*additional data* *long term F‐U*		TGCT OE	47	5.6 [2.3–23.1]	16.3 [8–28.8]	9.7 [3.6–34.7]	76 [10–187]				
		TGCT OE + CT	151	7.0 [1.9–37.5]	13.7 [7–32]	14.9 [2.4–80]	68 [28–151]				
					*Lower in OE + CT group*.						
**Murugaesu (2009)** ^16^	TGCT	OE with or without CT	39	6.9 < 5.0–13.5 > [0.3–80.1]	14.0 < 10.9–19.1 > [4.2–56.4]	12 < 6.4–27 > [0.9–149.2]	88 < 71.5–115 > [50–138]	31 < 26.8–35.3 > [10–70]	59 < 50–69 > [16–141]		
		OE	14	7.1 < 5.1–14.1 > [0.3–80.1]	13.0 < 9.9–14.9 > [4.2–56.4]	15.9 < 7.0–28.2 > [0.9–149.2]	88 < 73–111 > [50–120]	31 < 25.3–36.8 > [10–70]	59 < 46–72 > [16–122]		
		OE + CT	25	6.8 < 4.7–11.7 > [3.4–34.6]	17.4 < 13.9–25.3 > [7.8–28]	9.85 < 6.0–21.2 > [4.2–42.4]	92.5 < 67–127 > [53–138]	31 < 24.0–38.0 > [15–46]	59 < 40–79 > [19–141]		
				*NS*	*Higher in OE + CT group (p = 0.01)*	*NS*	*NS*		*NS*		
**Ondrusova (2009)** ^25^	TGCT	Full group	879								
	Unilateral disease		823	Elevated (>8.2 mU/ml) in 123 (15%)	Deficiency in 124 (15.1%)						
		OE + CT	NR	Elevated^g^							
		OE + RT	NR	Elevated^g^							
		OE + CT + RT	NR	Elevated^g^	Decreased^g^						
		RT in total	NR	Elevated^g^							
		CT in total	NR	Elevated^g^							
	Bilateral disease	All treatments	56	Elevated in 45 patients (83.9%)	deficiency in 47 patients (83.9%)						
**Brown (2006)** ^22^	TGCT	OE alone	101	6.98 (3.4)	12.0 (4.6)	13.6 (9.55)	25.5 (5.8)				36.2 (15.2)
		OE + CT	64	8.26 (6.21)	13.1 (7.7)	18.4 (14.4)	27 (8.4)				37 (17.2)
				*NS increase in CT group (p = 0.398)*	*NS difference, p = 0.663*	*Elevated in CT group (p = 0.034)*	*NS difference (p = 0.198)*				*NS difference*
**Stutz (1998)** ^27^	TGCT patients, intra‐patient comparison	full group	30								

Abbreviations: CT, chemotherapy; FSH, follicle stimulating hormone; FU, follow‐up; IQR, inter quartile range; LH, luteinizing hormone; n.d., not determined; NS, nonsignificant; OE, orchiectomy; PTH, para‐thyroid hormone; RT, radio therapy; SD, standard deviation; SHBG, sex hormone binding globulin; TGCT, testicular germ cell tumour; VF, vertebral fracture.

^a^ Only normo‐testosteronemic patients were evaluated in the study reported by Foresta et al. (2013) (testosterone normal‐range not reported).

^b^ LH reference ranges: Ondrusova 2018: <8.2 mU/ml, IJpma: not reported, Isaksson: 1.0–10.0 U/L, Willemse 2014:2.0–10.0 U/L, Foresta: not reported, Willemse 2010: 2.0–10.0 U/L, Murugaesu: 1.80–8.0 U/L, Ondrusova 2009:, Brown: 1.7–12.2 IU/L.

^c^ Testosterone reference ranges: Ondrusova 2018: >12.0 nmol/L, IJpma: not reported, Isaksson: <10 nmol/L, Willemse 2014: 8.0–35.0 nmol/L, Foresta: not reported, Willemse 2010: 8–35 nmol/L, Murugaesu: 9–27 nmol/L, Ondrusova 2009: 12.0–28.0 nmol/L, Brown: 7.1–24.1 nmol/L.

^d^ FSH reference ranges: Willemse 2014: 2.0–10.0 U/L, Foresta: not reported, Willemse 2010: 2.0–10.0 U/L, Murugaesu: 1.0–10.0 U/L, Brown: 2.0–18.1 IU/L.

^e^ Estradiol reference ranges: Willemse 2014: 70–200 pmol/L, Foresta: not reported, Willemse 2010: 70–200 pmol/L, Murugaesu: 28–156 pmol/L, Brown: 13.5–59.5 pg/ml.

^f^ SHBG reference ranges: Willemse 2010: 20–55 nmol/L, Isaksson: 13–90 nmol/L, Willemse 2010: 20–55 nmol/L, Murugaesu: 17–50 nmol/L.

^g^ These outcomes were reported to be statistically significant increase or decrease, but *p* values, means and SD or medians and ranges could not be retrieved.

Subgroups of TGCT patients were found to have an increased LH level in six studies, of which five studies reported a significant difference specifically between treatment groups (chemotherapy or patients/controls).^21^, ^23^, ^24^, ^25^, ^26^ Willemse (2014) reported higher LH levels and lower BMD at follow‐up in patients with more advanced (disseminated) TGCT compared to stage I TGCT.^20^ Significantly increased LH was found in combination with a significantly lower BMD in five out of six studies,^20^, ^21^, ^23^, ^25^, ^26^ Isaksson, who also reported increased LH levels, found a non‐significant decrease in BMD.^24^ Willemse (2010), Murugaesu and Brown found no significant changes in LH and also no difference in BMD outcomes.

Four studies reported significantly lower testosterone levels in TCGT. Willemse 2010, Ondrusova 2009 and Ondrusova 2018 showed significantly lower serum free testosterone levels 3 months to 30 years after treatment in patients who had undergone orchiectomy and chemotherapy, compared to those who had undergone orchiectomy alone.^15^, ^25^, ^26^ IJpma reported free testosterone levels were significantly lower in TGCT patients at follow‐up, compared to levels in healthy volunteers and they simultaneously reported a lower BMD.^21^ Murugaesu reported higher levels of free testosterone in the orchiectomy and chemotherapy group associated with a higher BMD compared to patients who had orchiectomy alone.^16^ The other four studies which reported on testosterone levels did not report significant or clinically relevant differences or trends.^20^, ^22^, ^23^, ^24^

Estradiol levels were measured in five studies. Willemse (2014) reported significantly higher pretreatment estradiol levels (*p* = 0.007) in patients with disseminated disease, compared with levels in those with stage 1 disease.^20^ In the other four studies no significant differences were found.^15^, ^16^, ^22^, ^23^

Plasma concentrations of follicle stimulating hormone (FSH) were reported in five studies.^15^, ^16^, ^20^, ^22^, ^23^ Significantly higher FSH levels were found in TGCT patients compared to patients with sexual dysfunction by Foresta.^23^ In addition, Willemse (2010 and 2014) and Brown reported higher FSH levels in subgroups of patients with disseminated TGCT after chemotherapy, or after a longer follow‐up.^15^, ^20^, ^22^ Murugaesu did not report significant differences in FSH levels between treatment groups, nor differences in BMD between groups.

Vitamin D and parathyroid hormone levels were measured in four studies.^15^, ^16^, ^22^, ^23^ Except Foresta (low PTH and Vitamin D, *p* < 0.00001), no significant differences were found. No statistically significant differences were found in plasma levels of calcium or sex hormone binding globulin (SHBG) in any of the studies included.

## 4 DISCUSSION

TGCT survivors, particularly those treated with chemotherapy, are at increased risk of having a low BMD. Evidence for this is mainly provided by data generated from two robust longitudinal studies showing a lower BMD in TGCT patients treated with chemotherapy compared to TGCT patients treated with orchiectomy only.^20^, ^21^ A second risk factor for decreased BMD, identified in these patients is a long duration of follow‐up, also after correction for age,^20^, ^21^, ^23^, ^25^ possibly due to long‐term effects of chemotherapy, the cumulative dose of corticosteroids administered as antiemetic treatment during chemotherapy, or longer exposure to hypogonadism.^6^, ^9^ High serum LH concentrations were found to be associated with low BMD measurements, also in the absence of low serum testosterone levels,^20^, ^23^, ^24^ suggesting that LH may have a direct negative effect on bone remodelling, representing an independent risk factor for osteoporosis. This, however, remains to be established, as most studies did not include a separate analysis of the effect of gonadal status on BMD outcomes, which may identify the groups most at risk. The finding of high LH rather than low testosterone in TGCT survivors is in line with findings of three other studies which did not show a relationship between serum estradiol and bone health or fracture risk.^6^, ^7^, ^28^ Use of corticosteroids was not reported in half of the studies and none of the studies performed a separate analysis or reported the dose/duration of corticosteroid treatment.^20^, ^29^

The only study systematically addressing the skeletal complications of TGCT in long‐term survivors revealed a high prevalence of radiologically diagnosed, often asymptomatic, vertebral fractures pointing to an increased fracture risk, even in the absence of a low BMD.^15^ Findings from this study thus suggest that it is not only a decrease in bone quantity but potentially also a decrease in bone quality that may be responsible for the increased fracture risk observed in TGCT patients. Whether this fracture risk could be decreased or prevented by bone modifying treatment remains to be established.

This review has strengths as well as limitations. Its main strength is that to our knowledge, this is the first review that provides a complete overview of the current, albeit scarce literature on bone health, fracture risk and potential risk factors associated with loss of bone mass and increased fracture risk in TGCT survivors. A further strength of this review is that it is a PRISMA‐adhering systematic review using a robust summation of available evidence on bone health in TGCT survivors.

The review also has a number of limitations, including the heterogeneity and risk of bias of the populations studied and of reported outcomes, the small number of patients included in each study (mostly <100 patients) and the inability to access individual data for most studies, thus precluding the conduct of a meta‐analysis. Eight of the 10 studies included in the review had a non‐randomized, retrospective design, and the remaining two were non‐randomized prospective studies.^20^, ^21^ Some studies also used different measurement devices, not cross‐calibrated with each other and used at different time windows with different reference values.^30^, ^31^, ^32^ These limitations highlight the need for standardized protocols, the collection of full sets of data and uniform methods of reporting in order to allow the issuing of best clinical guidelines and recommendations on how to best manage the skeletal complications of TGCT.

### 4.1 Implications for clinical practice

Despite the scarce data available, findings from this systematic review of the literature reinforce the view that bone health, especially fracture risk should be thoroughly evaluated and monitored in newly diagnosed as well as long‐term TGCT survivors, an unmet need not addressed by the current, recently updated (2021) EAU guideline for follow‐up of germ cell tumour survivors.^17^ The 2014 Endocrine Society's guidelines for the diagnosis of osteoporosis in men recommends screening hypogonadal men for osteoporosis from the age of 50.^33^ However, TGCT survivors are generally young and survival rates have significantly improved, so that they might be exposed to the long‐term effects of chronic hypogonadism, further increasing their future risk for osteoporosis, fragility fractures and associated morbidities.^1^, ^2^, ^31^, ^34^ However, data are still scarce in this field and further research is warranted to reach firmer conclusions on the relationship between treatment modalities, hypogonadism, BMD outcomes and fracture risk in TCGC survivors. Notwithstanding, in keeping with findings reported in studies included in this systematic review showing a high prevalence of abnormal gonadal status in TGCT patients that may significantly impact on bone health, we would urge for special attention to be paid to the evaluation and monitoring of gonadal hormone status and bone health including BMD measurements and clinical and radiological evaluation of fracture risk in newly diagnosed as well as long‐term survivors of this malignancy regardless of their age.^33^, ^34^

### 4.2 Implications for future research

In addition to the systematic collection of data, using standardized protocols for consolidation of the scarce available evidence, several additional issues remain to be explored on the pathophysiology of decrease bone quantity and/bone quality in TGCT survivors, both being potentially associated with increased bone fragility. There is an unmet need to address fracture rates in all future studies on TGCT survivors as solid fracture outcome data are lacking in the majority of thus far reported studies. Potential areas of interest include the role of abnormalities in gonadal hormones and in Leydig cell function, the latter reported to be prevalent in 9%–27% of TGCT patients.^6^, ^7^, ^35^ On this topic, it would be of potential value to explore the value of human chorionic gonadotropin (hCG) levels as a biomarker of pituitary‐Leydig cell axis function, in identifying patients at risk of developing hypogonadism‐related complications.^36^

### 4.3 Conclusions

Despite high risk of bias in all included studies, our findings from this systematic review suggest that TGCT survivors are at risk for skeletal complications in the form of decreased bone mass and increased bone fragility, also independently from BMD. Risk factors identified are chemotherapy‐associated abnormalities in gonadal status and longer survival. These findings call for gonadal hormone status and bone health (including BMD) measurements and clinical and radiological evaluation of fracture risk to be investigated and monitored in newly diagnosed as well as long‐term survivors of this malignancy regardless of age, in order to enable early diagnosis and management to reverse or prevent these complications.

## CONFLICT OF INTEREST

ICMJE disclosure forms submitted. J.P.M. Vrouwe has no conflict of interest to declare. P.M.L. Hennus has no conflict of interest to declare. N.A.T. Hamdy has no conflict of interest to declare. S. Osanto has no conflict of interest to declare. P.M. Willemse has no conflict of interest to declare.

## AUTHOR CONTRIBUTION

J.P.M. Vrouwe: methodology, investigation, data curation, writing‐original draft, visualization and administration; P.M.L. Hennus: methodology, investigation and writing‐review and editing; N.A.T. Hamdy: writing‐review and editing; S. Osanto: conceptualization, methodology and writing‐review; P.M. Willemse: conceptualization, investigation, methodology, writing‐review and supervision.

## Supporting information


**Data S1** Supporting Information
