# Supplementary material for: Risk of osteoporosis in testicular germ cell tumour survivors: A systematic review of the literature
Source: BJUI Compass. 2022 Aug 18;4(1):24–43. doi: 10.1002/bco2.183 (PMC9766871; doi:10.1002/bco2.183)
Supplement: Supplementary file 1 — Data S1 Supporting Information [file BCO2-4-24-s001.docx]

**Supplementary materials**

**Search strategy**

Databases: PubMed, MedLine, Embase, Web of Science, Cochrane, Central, Emcare, PeDRO, PsychINFO, ERIC, Academic Search Premier

**PubMed**

<http://www.ncbi.nlm.nih.gov/pubmed?otool=leiden>

3-1-2019

(("Osteoporosis"[Mesh] OR "osteoporosis"[tw] OR "Osteoporotic Fractures"[Mesh] OR osteopor*[tw] OR "fragility fracture"[tw] OR "fragility fractures"[tw] OR (fragilit*[tw] AND fractur*[tw]) OR "bone mineral density"[tw] OR "bone density"[tw] OR "Bone Density"[mesh] OR "bone metabolism"[tw] OR "Bone and Bones/metabolism"[mesh] OR "bone health"[tw] OR "skeletal health"[tw] OR "bone damage"[tw] OR "skeletal damage"[tw] OR "Bone and Bones/drug effects"[mesh] OR "Bone Diseases, Metabolic"[mesh] OR "Fractures, Bone"[Mesh] OR "bone fracture"[tw] OR "bone fractures"[tw] OR fractur*[tw]) AND ("Testicular Neoplasms"[Mesh] OR "testicular neoplasm"[tw] OR "testicular neoplasms"[tw] OR "testicular cancer"[tw] OR "testicular cancers"[tw] OR "testicular germ cell cancer"[tw] OR "testicular germ cell cancers"[tw] OR "testicular carcinoma"[tw] OR "testicular carcinomas"[tw] OR "testicular adenocarcinoma"[tw] OR "testicular tumor"[tw] OR "testicular tumors"[tw] OR "testicular germ cell tumor"[tw] OR "testicular germ cell tumors"[tw] OR "testicular tumour"[tw] OR "testicular tumours"[tw] OR "testicular germ cell tumour"[tw] OR "testicular germ cell tumours"[tw] OR "testicular malignancy"[tw] OR "testicular malignancies"[tw] OR "testis neoplasm"[tw] OR "testis neoplasms"[tw] OR "testis cancer"[tw] OR "testis cancers"[tw] OR "testis carcinoma"[tw] OR "testis carcinomas"[tw] OR "testis adenocarcinoma"[tw] OR "testis tumor"[tw] OR "testis tumors"[tw] OR "testis tumour"[tw] OR "testis tumours"[tw] OR "testis malignancy"[tw] OR "testicle cancer"[tw] OR "testicle tumors"[tw] OR "testicle tumour"[tw] OR "testicle tumours"[tw] OR "cancer of testis"[tw] OR "cancer of the testis"[tw] OR "carcinoma of the testis"[tw] OR "tumor of testis"[tw] OR "tumor of the testis"[tw] OR "tumour of testis"[tw] OR "tumour of the testis"[tw] OR (("testicular"[tw] OR "testis"[tw] OR "testes"[tw] OR "testicle"[tw] OR "testicles"[tw] OR testic*[tw]) AND ("neoplasm"[tw] OR "neoplasms"[tw] OR "cancer"[tw] OR "cancers"[tw] OR "carcinoma"[tw] OR "carcinomas"[tw] OR "adenocarcinoma"[tw] OR "tumor"[tw] OR "tumors"[tw] OR "tumour"[tw] OR "tumours"[tw] OR "malignancy"[tw] OR "malignancies"[tw] OR malignan*[tw])))) NOT ("Animals"[mesh] NOT "Humans"[mesh])

**MEDLINE**

<http://gateway.ovid.com/ovidweb.cgi?T=JS&MODE=ovid&NEWS=n&PAGE=main&D=prmz>

**Embase**

<http://ovidsp.ovid.com/ovidweb.cgi?T=JS&PAGE=main&MODE=ovid&D=oemezd>

((exp *"Osteoporosis"/ OR "osteoporosis".ti,ab OR "Fragility Fracture"/ OR osteopor*.ti,ab OR "fragility fracture".ti,ab OR "fragility fractures".ti,ab OR (fragilit*.ti,ab ADJ4 fractur*.ti,ab) OR "bone mineral density".ti,ab OR "bone density".ti,ab OR exp *"Bone Density"/ OR "bone metabolism".ti,ab OR exp *"Bone Metabolism"/ OR "bone health".ti,ab OR "skeletal health".ti,ab OR "bone damage".ti,ab OR "skeletal damage".ti,ab OR exp *"Bone"/an OR exp *"metabolic bone disease"/ OR exp *"Bone Injury"/ OR "bone fracture".ti,ab OR "bone fractures".ti,ab OR fractur*.ti,ab **OR "frax".ti,ab** **OR *"Bone Remodeling"/ OR "Bone Remodeling".ti,ab OR "Bone Remodelling".ti,ab OR "Bone Turnover".ti,ab OR "Bone Regeneration".ti,ab OR "Osseointegration".ti,ab OR "Bone Resorption".ti,ab OR *"Osteolysis"/ OR "Osteolysis".ti,ab**) AND (exp *"Testis Tumor"/ OR "testicular neoplasm".ti,ab OR "testicular neoplasms".ti,ab OR "testicular cancer".ti,ab OR "testicular cancers".ti,ab OR "testicular germ cell cancer".ti,ab OR "testicular germ cell cancers".ti,ab OR "testicular carcinoma".ti,ab OR "testicular carcinomas".ti,ab OR "testicular adenocarcinoma".ti,ab OR "testicular tumor".ti,ab OR "testicular tumors".ti,ab OR "testicular germ cell tumor".ti,ab OR "testicular germ cell tumors".ti,ab OR "testicular tumour".ti,ab OR "testicular tumours".ti,ab OR "testicular germ cell tumour".ti,ab OR "testicular germ cell tumours".ti,ab OR "testicular malignancy".ti,ab OR "testicular malignancies".ti,ab OR "testis neoplasm".ti,ab OR "testis neoplasms".ti,ab OR "testis cancer".ti,ab OR "testis cancers".ti,ab OR "testis carcinoma".ti,ab OR "testis carcinomas".ti,ab OR "testis adenocarcinoma".ti,ab OR "testis tumor".ti,ab OR "testis tumors".ti,ab OR "testis tumour".ti,ab OR "testis tumours".ti,ab OR "testis malignancy".ti,ab OR "testicle cancer".ti,ab OR "testicle tumors".ti,ab OR "testicle tumour".ti,ab OR "testicle tumours".ti,ab OR "cancer of testis".ti,ab OR "cancer of the testis".ti,ab OR "carcinoma of the testis".ti,ab OR "tumor of testis".ti,ab OR "tumor of the testis".ti,ab OR "tumour of testis".ti,ab OR "tumour of the testis".ti,ab OR (("testicular".ti,ab OR "testis".ti,ab OR "testes".ti,ab OR "testicle".ti,ab OR "testicles".ti,ab OR testic*.ti,ab) ADJ5 ("neoplasm".ti,ab OR "neoplasms".ti,ab OR "cancer".ti,ab OR "cancers".ti,ab OR "carcinoma".ti,ab OR "carcinomas".ti,ab OR "adenocarcinoma".ti,ab OR "tumor".ti,ab OR "tumors".ti,ab OR "tumour".ti,ab OR "tumours".ti,ab OR "malignancy".ti,ab OR "malignancies".ti,ab OR malignan*.ti,ab)))) AND exp "Humans"/

the "syn" command

NOT (conference review or conference abstract).pt

**Web of Science**

<http://isiknowledge.com/wos>

(ts=("Osteoporosis" OR "osteoporosis" OR "Fragility Fracture" OR osteopor* OR "fragility fracture" OR "fragility fractures" OR (fragilit* NEAR4 fractur*) OR "bone mineral density" OR "bone density" OR "Bone Density" OR "bone metabolism" OR "Bone Metabolism" OR "bone health" OR "skeletal health" OR "bone damage" OR "skeletal damage" OR "metabolic bone disease" OR "Bone Injury" OR "bone fracture" OR "bone fractures" OR fractur* **OR "frax"** **OR "Bone Remodeling" OR "Bone Remodeling" OR "Bone Remodelling" OR "Bone Turnover" OR "Bone Regeneration" OR "Osseointegration" OR "Bone Resorption" OR "Osteolysis" OR "Osteolysis"**) AND ts=("Testis Tumor" OR "testicular neoplasm" OR "testicular neoplasms" OR "testicular cancer" OR "testicular cancers" OR "testicular germ cell cancer" OR "testicular germ cell cancers" OR "testicular carcinoma" OR "testicular carcinomas" OR "testicular adenocarcinoma" OR "testicular tumor" OR "testicular tumors" OR "testicular germ cell tumor" OR "testicular germ cell tumors" OR "testicular tumour" OR "testicular tumours" OR "testicular germ cell tumour" OR "testicular germ cell tumours" OR "testicular malignancy" OR "testicular malignancies" OR "testis neoplasm" OR "testis neoplasms" OR "testis cancer" OR "testis cancers" OR "testis carcinoma" OR "testis carcinomas" OR "testis adenocarcinoma" OR "testis tumor" OR "testis tumors" OR "testis tumour" OR "testis tumours" OR "testis malignancy" OR "testicle cancer" OR "testicle tumors" OR "testicle tumour" OR "testicle tumours" OR "cancer of testis" OR "cancer of the testis" OR "carcinoma of the testis" OR "tumor of testis" OR "tumor of the testis" OR "tumour of testis" OR "tumour of the testis" OR (("testicular" OR "testis" OR "testes" OR "testicle" OR "testicles" OR testic*) ADJ5 ("neoplasm" OR "neoplasms" OR "cancer" OR "cancers" OR "carcinoma" OR "carcinomas" OR "adenocarcinoma" OR "tumor" OR "tumors" OR "tumour" OR "tumours" OR "malignancy" OR "malignancies" OR malignan*)))) **NOT ti=("veterinary" OR "rabbit" OR "rabbits" OR "animal" OR "animals" OR "mouse" OR "mice" OR "rodent" OR "rodents" OR "rat" OR "rats" OR "pig" OR "pigs" OR "porcine" OR "horse" OR "horses" OR "equine" OR "cow" OR "cows" OR "bovine" OR "goat" OR "goats" OR "sheep" OR "ovine" OR "canine" OR "dog" OR "dogs" OR "feline" OR "cat" OR "cats")**

**Cochrane**

<http://www.cochranelibrary.com/>

(("Osteoporosis" OR "osteoporosis" OR "Fragility Fracture" OR osteopor* OR "fragility fracture" OR "fragility fractures" OR (fragilit* NEAR4 fractur*) OR "bone mineral density" OR "bone density" OR "Bone Density" OR "bone metabolism" OR "Bone Metabolism" OR "bone health" OR "skeletal health" OR "bone damage" OR "skeletal damage" OR "metabolic bone disease" OR "Bone Injury" OR "bone fracture" OR "bone fractures" OR fractur* **OR "frax"** **OR "Bone Remodeling" OR "Bone Remodeling" OR "Bone Remodelling" OR "Bone Turnover" OR "Bone Regeneration" OR "Osseointegration" OR "Bone Resorption" OR "Osteolysis" OR "Osteolysis"**) AND ("Testis Tumor" OR "testicular neoplasm" OR "testicular neoplasms" OR "testicular cancer" OR "testicular cancers" OR "testicular germ cell cancer" OR "testicular germ cell cancers" OR "testicular carcinoma" OR "testicular carcinomas" OR "testicular adenocarcinoma" OR "testicular tumor" OR "testicular tumors" OR "testicular germ cell tumor" OR "testicular germ cell tumors" OR "testicular tumour" OR "testicular tumours" OR "testicular germ cell tumour" OR "testicular germ cell tumours" OR "testicular malignancy" OR "testicular malignancies" OR "testis neoplasm" OR "testis neoplasms" OR "testis cancer" OR "testis cancers" OR "testis carcinoma" OR "testis carcinomas" OR "testis adenocarcinoma" OR "testis tumor" OR "testis tumors" OR "testis tumour" OR "testis tumours" OR "testis malignancy" OR "testicle cancer" OR "testicle tumors" OR "testicle tumour" OR "testicle tumours" OR "cancer of testis" OR "cancer of the testis" OR "carcinoma of the testis" OR "tumor of testis" OR "tumor of the testis" OR "tumour of testis" OR "tumour of the testis" OR (("testicular" OR "testis" OR "testes" OR "testicle" OR "testicles" OR testic*) ADJ5 ("neoplasm" OR "neoplasms" OR "cancer" OR "cancers" OR "carcinoma" OR "carcinomas" OR "adenocarcinoma" OR "tumor" OR "tumors" OR "tumour" OR "tumours" OR "malignancy" OR "malignancies" OR malignan*)))):ti,ab,kw

**CENTRAL**

<https://archie.cochrane.org/index.jsp?redirectTo=http://crso.cochrane.org/login.php&key=58c18a4283f80>

schoones/

**Emcare** <http://ovidsp.ovid.com/ovidweb.cgi?T=JS&NEWS=n&CSC=Y&PAGE=main&D=emcr>

**(**((exp "Osteoporosis"/ OR "osteoporosis".mp OR "Fragility Fracture"/ OR osteopor*.mp OR "fragility fracture".mp OR "fragility fractures".mp OR (fragilit*.mp ADJ4 fractur*.mp)) AND (exp "Testis Tumor"/ OR "testicular neoplasm".mp OR "testicular neoplasms".mp OR "testicular cancer".mp OR "testicular cancers".mp OR "testicular germ cell cancer".mp OR "testicular germ cell cancers".mp OR "testicular carcinoma".mp OR "testicular carcinomas".mp OR "testicular adenocarcinoma".mp OR "testicular tumor".mp OR "testicular tumors".mp OR "testicular germ cell tumor".mp OR "testicular germ cell tumors".mp OR "testicular tumour".mp OR "testicular tumours".mp OR "testicular germ cell tumour".mp OR "testicular germ cell tumours".mp OR "testicular malignancy".mp OR "testicular malignancies".mp OR "testis neoplasm".mp OR "testis neoplasms".mp OR "testis cancer".mp OR "testis cancers".mp OR "testis carcinoma".mp OR "testis carcinomas".mp OR "testis adenocarcinoma".mp OR "testis tumor".mp OR "testis tumors".mp OR "testis tumour".mp OR "testis tumours".mp OR "testis malignancy".mp OR "testicle cancer".mp OR "testicle tumors".mp OR "testicle tumour".mp OR "testicle tumours".mp OR "cancer of testis".mp OR "cancer of the testis".mp OR "carcinoma of the testis".mp OR "tumor of testis".mp OR "tumor of the testis".mp OR "tumour of testis".mp OR "tumour of the testis".mp OR (("testicular".mp OR "testis".mp OR "testes".mp OR "testicle".mp OR "testicles".mp OR testic*.mp) ADJ5 ("neoplasm".mp OR "neoplasms".mp OR "cancer".mp OR "cancers".mp OR "carcinoma".mp OR "carcinomas".mp OR "adenocarcinoma".mp OR "tumor".mp OR "tumors".mp OR "tumour".mp OR "tumours".mp OR "malignancy".mp OR "malignancies".mp OR malignan*.mp)))) **OR** (("bone mineral density".ti,ab OR "bone density".ti,ab OR *"Bone Density"/ OR "bone metabolism".ti,ab OR exp *"Bone metabolism"/ OR "bone health".ti,ab OR "skeletal health".ti,ab OR "bone damage".ti,ab OR "skeletal damage".ti,ab OR exp *"Metabolic Bone Disease"/ OR exp *"Fracture"/ OR "bone fracture".ti,ab OR "bone fractures".ti,ab OR fractur*.ti,ab) AND (exp *"Testis Tumor"/ OR "testicular neoplasm".ti,ab OR "testicular neoplasms".ti,ab OR "testicular cancer".ti,ab OR "testicular cancers".ti,ab OR "testicular germ cell cancer".ti,ab OR "testicular germ cell cancers".ti,ab OR "testicular carcinoma".ti,ab OR "testicular carcinomas".ti,ab OR "testicular adenocarcinoma".ti,ab OR "testicular tumor".ti,ab OR "testicular tumors".ti,ab OR "testicular germ cell tumor".ti,ab OR "testicular germ cell tumors".ti,ab OR "testicular tumour".ti,ab OR "testicular tumours".ti,ab OR "testicular germ cell tumour".ti,ab OR "testicular germ cell tumours".ti,ab OR "testicular malignancy".ti,ab OR "testicular malignancies".ti,ab OR "testis neoplasm".ti,ab OR "testis neoplasms".ti,ab OR "testis cancer".ti,ab OR "testis cancers".ti,ab OR "testis carcinoma".ti,ab OR "testis carcinomas".ti,ab OR "testis adenocarcinoma".ti,ab OR "testis tumor".ti,ab OR "testis tumors".ti,ab OR "testis tumour".ti,ab OR "testis tumours".ti,ab OR "testis malignancy".ti,ab OR "testicle cancer".ti,ab OR "testicle tumors".ti,ab OR "testicle tumour".ti,ab OR "testicle tumours".ti,ab OR "cancer of testis".ti,ab OR "cancer of the testis".ti,ab OR "carcinoma of the testis".ti,ab OR "tumor of testis".ti,ab OR "tumor of the testis".ti,ab OR "tumour of testis".ti,ab OR "tumour of the testis".ti,ab OR (("testicular".ti,ab OR "testis".ti,ab OR "testes".ti,ab OR "testicle".ti,ab OR "testicles".ti,ab OR testic*.ti,ab) ADJ5 ("neoplasm".ti,ab OR "neoplasms".ti,ab OR "cancer".ti,ab OR "cancers".ti,ab OR "carcinoma".ti,ab OR "carcinomas".ti,ab OR "adenocarcinoma".ti,ab OR "tumor".ti,ab OR "tumors".ti,ab OR "tumour".ti,ab OR "tumours".ti,ab OR "malignancy".ti,ab OR "malignancies".ti,ab OR malignan*.ti,ab))))**)** AND exp "Humans"/

**PeDRO**

<http://www.pedro.org.au/>

**PsycINFO**

<http://search.ebscohost.com/login.aspx?authtype=ip,uid&profile=lumc&defaultdb=psyh>

**ERIC**

<https://eric.ed.gov/>

Via Ebsco**:** <http://databases.library.leiden.edu/?bibid=990024848320302711&redirect=true>

Via OVID: <http://ovidsp.tx.ovid.com/sp-3.21.0a/ovidweb.cgi?&S=FKFBFPLFBNDDFEMKNCIKKBGCMKJJAA00&New+Database=Single%7c4>

**Academic Search Premier [fulltextzoeken]**

<http://search.ebscohost.com/login.aspx?authtype=ip,uid&profile=lumc&defaultdb=aph>

TI/SU/AB/KW

(("Osteoporosis" OR "osteoporosis" OR "Fragility Fracture" OR osteopor* OR "fragility fracture" OR "fragility fractures" OR (fragilit* NEAR4 fractur*) OR "bone mineral density" OR "bone density" OR "Bone Density" OR "bone metabolism" OR "Bone Metabolism" OR "bone health" OR "skeletal health" OR "bone damage" OR "skeletal damage" OR "metabolic bone disease" OR "Bone Injury" OR "bone fracture" OR "bone fractures" OR fractur* **OR "frax"** **OR "Bone Remodeling" OR "Bone Remodeling" OR "Bone Remodelling" OR "Bone Turnover" OR "Bone Regeneration" OR "Osseointegration" OR "Bone Resorption" OR "Osteolysis" OR "Osteolysis"**) AND ("Testis Tumor" OR "testicular neoplasm" OR "testicular neoplasms" OR "testicular cancer" OR "testicular cancers" OR "testicular germ cell cancer" OR "testicular germ cell cancers" OR "testicular carcinoma" OR "testicular carcinomas" OR "testicular adenocarcinoma" OR "testicular tumor" OR "testicular tumors" OR "testicular germ cell tumor" OR "testicular germ cell tumors" OR "testicular tumour" OR "testicular tumours" OR "testicular germ cell tumour" OR "testicular germ cell tumours" OR "testicular malignancy" OR "testicular malignancies" OR "testis neoplasm" OR "testis neoplasms" OR "testis cancer" OR "testis cancers" OR "testis carcinoma" OR "testis carcinomas" OR "testis adenocarcinoma" OR "testis tumor" OR "testis tumors" OR "testis tumour" OR "testis tumours" OR "testis malignancy" OR "testicle cancer" OR "testicle tumors" OR "testicle tumour" OR "testicle tumours" OR "cancer of testis" OR "cancer of the testis" OR "carcinoma of the testis" OR "tumor of testis" OR "tumor of the testis" OR "tumour of testis" OR "tumour of the testis" OR (("testicular" OR "testis" OR "testes" OR "testicle" OR "testicles" OR testic*) ADJ5 ("neoplasm" OR "neoplasms" OR "cancer" OR "cancers" OR "carcinoma" OR "carcinomas" OR "adenocarcinoma" OR "tumor" OR "tumors" OR "tumour" OR "tumours" OR "malignancy" OR "malignancies" OR malignan*))))

**Social Services Abstracts**

<https://search.proquest.com/socialservices?accountid=12045>

<http://databases.library.leiden.edu/?bibid=990027333030302711&redirect=true>

**Sociological Abstracts**

<https://search.proquest.com/socabs/index>

<http://databases.library.leiden.edu/?bibid=990024621960302711&redirect=true>

**Trials**

**WHO International Clinical Trials Registry Platform**

<http://apps.who.int/trialsearch/>

**ClinicalTrials.gov**

<http://clinicaltrials.gov/>

+ beta-site: <https://clinicaltrials.gov/beta/>

**European Union Clinical Trials Register**

<https://www.clinicaltrialsregister.eu/ctr-search/search>

**Current Controlled Trials**

<http://www.controlled-trials.com/>

**Multi-register**

<http://www.controlled-trials.com/mrct/>

**Open Trials**

<http://explorer.opentrials.net/search?q>

**Cochrane**

<http://www.cochranelibrary.com/>

**CENTRAL**

<https://archie.cochrane.org/index.jsp?redirectTo=http://crso.cochrane.org/login.php&key=58c18a4283f80>

schoones/

**Systematic Reviews**

**Prospero**

<http://www.crd.york.ac.uk/prospero/>

**Epistemonikos**

<https://www.epistemonikos.org/en/advanced_search>

**Cochrane**

<http://www.cochranelibrary.com/>

**CENTRAL**

<https://archie.cochrane.org/index.jsp?redirectTo=http://crso.cochrane.org/login.php&key=58c18a4283f80>

**PubMed**

<https://www.ncbi.nlm.nih.gov/pubmed/?term=systematic%5Bsb%5D>

**Converis**

<https://converis.lumc.nl/converis/secure/client/login>

Journal databases [fulltextsearch]

**A. ScienceDirect**

<http://www.sciencedirect.com/science?_ob=MiamiSearchURL&_method=requestForm&_temp=all_boolSearch.tmpl&_acct=C000026638&_version=1&_urlVersion=1&_userid=530453&md5=d44bd9fa9076bb9b258a588b309be1e3>

TITLE-ABSTR-KEY**()**

**B. Springer**

<http://link.springer.com/advanced-search>

**C. Wiley**

<http://onlinelibrary.wiley.com/advanced/search>

**D. LWW**

<http://ovidsp.ovid.com/ovidweb.cgi?T=JS&PAGE=main&MODE=ovidclassic&D=ovft>

**~~E. Highwire [gestopt per 1/2017]~~**

[~~http://highwire.stanford.edu/cgi/search~~](http://highwire.stanford.edu/cgi/search)

**F. Taylor & Francis/Informaworld**

<https://www.tandfonline.com/>

**G. Oxford University Press - Oxford Index**

<http://oxfordindex.oup.com/>

**H. Cambridge Core**

<https://www.cambridge.org/core>

**I. Google Scholar**

<http://scholar.google.com/>

**J. Microsoft Academic Search**

<http://academic.research.microsoft.com/>

**K. Open Access Library**

<http://www.oalib.com/>

**L. Europe PMC**

<http://europepmc.org/advancesearch>

**M. Canada PMC**

<http://pubmedcentralcanada.ca/pmcc/>

**N. PMC**

<http://www.ncbi.nlm.nih.gov/pmc/>

**O. MedNar**

<http://mednar.com/mednar/>

**P. WorldWideScience**

<http://worldwidescience.org/>

**Q. DeepDyve**

<http://www.deepdyve.com/>

**R. Bielefeld Academic Search Engine**

<http://base-search.net/>

**S. eBook Collection (EBSCOhost)**

<http://digitallibrary.leidenuniv.nl/V?func=native-link&resource=LDN12394>

**___________________________________________________________________**

**MeSH on Demand:**

<http://ii.nlm.nih.gov/Interactive/MeSHonDemand.shtml>

**PubMed-check:**

<http://buildsucceeded.nl/pubmed/>

..ddup x

docz.dz

96xPiFQH

**Deduplication**: <http://crebp-sra.com/>

<http://buildsucceeded.net/pubmed/>

MeSH-Browser <https://meshb.nlm.nih.gov/#/fieldSearch>

PubMed Health - <https://www.ncbi.nlm.nih.gov/pubmedhealth/>

[<http://mesh.med.yale.edu/>]
